# Comparison of different New Radio (NR) waveforms for wireless communications

**DOI:** 10.1371/journal.pone.0283886

**Published:** 2023-04-03

**Authors:** A. K. M. Baki

**Affiliations:** Department of EEE, Ahsanullah University of Science and Technology (AUST), Dhaka, Bangladesh; CESi Engineering School: Ecole d’Ingenieurs CESi, FRANCE

## Abstract

New Radio (NR) waveforms of existing wireless communication systems need further improvement in order to support future wireless communications. NR is the radio interface technology proposed by the 3^rd^ Generation Partnership Project (3GPP) for 5G. Prototype Filter (PF) of NR plays a vital role in performance improvement of wireless systems. NR waveforms can adapt in a better way to different channel conditions. Some of the NR filtering techniques are Filtered-OFDM (F-OFDM), Filter Bank Multi-Carrier (FBMC), and Universal Filtered Multi-Carrier (UFMC). NR waveforms require performance improvement when high reliability, massive connectivity, lower power consumption, and time-critical applications are required. Areas of improvement are Power Spectral Density (PSD), Bit Error Rate (BER), Signal to Interference Ratio (SIR), Doppler Diversity, and Peak to Average Power Ratio (PAPR). This paper compares different performance parameters of Filtered-OFDM, FBMC, and UFMC using existing proto-type filters and novel proto-type filters. The novel and better PFs, described in the paper, were proposed first time by the authors and his research group. Proposed novel prototype filters for FBMC, Filtered-OFDM, and UFMC are respectively Binomial filter and Fractional Powered Binomial Filter (FPBF). With FPBF based OFDM, PSD improvement was 97.5 dB, and BER improvement was 0.07 at 0 dB SNR. With Binomial filter based FBMC, OOBE improvement was 19.7 dB and BER improvement was 0.03 at 0 dB SNR. PAPR improvement with Binomial filter based FBMC was 1.16 dB at 64-QAM and 1.1 dB at 256-QAM. With FPBF based UFMC, improvement of interference level was 122 dB within 3^rd^~52^th^ sub-bands due to 1^st^ sub-band. BER improvement was 0.09 at 0 dB SNR. SIR improvement was 5. 27 dB with 15 KHz sub-carrier spacing and 16.55 dB with 30 KHz sub-carrier spacing of UFMC. Novel NR filters, discussed in the paper, are good candidates for future 6G wireless systems.

## Introduction

‘New Radio (NR) is the radio technology proposed by 3^rd^ Generation Partnership Project (3GPP) for 5G communications’. NR uses OFDM-based waveforms.NR waveforms are more adaptable to channel conditions. This adaptability can be achieved through different sub-bands, sub-carrier spacing, and filtering/windowing techniques. Present wireless systems have some drawbacks such as higher Out Of Band Emission (OOBE), higher latency, and low reliability. As an alternative, NR air-interface was proposed in 5G to overcome the issues. Wireless systems require higher quality for some services at greater ranges, such as Machine Type Communications (MTC), Internet of Things (IoT), autonomous vehicular technologies etc. Three scenarios have been defined by ITU-R for IMT-2020 and beyond [1–4]:

1. eMBB (enhanced Mobile Broad Band),
2. mMTC (massive Machine Type Communications), and
3. uRLLC (ultra-Reliable and Low Latency Communications).

Internet of Things (IoT) comprise of billions of miscellaneous devices. ‘Increased integration of cellular and Wi-Fi standards’ will provide a ubiquitous, high-rate, low-latency experience for network users [4, 5]. Capacity of 5G wireless networks is 1000 times higher than that of 4G networks. Major five (05) technologies are deployed through present wireless communication systems such as:

1. millimeter-Wave (mmWave) band,
2. Massive Multi Input Multi Output (MIMO) techniques,
3. deployment of small cells,
4. beamforming techniques, and
5. Full-Duplex system.

Wireless systems should support high mobility users [6] or Mission Critical Communication (MCM) systems [1]. It is necessary to consider Doppler Effect for high mobility systems as well as low latency applications. Higher values of Signal to Interference Ratio (SIR) can improve Doppler Effect. Future 6G wireless systems will require even better performance.

Different NR waveforms have been described in different literatures. Some of the waveforms are:

1. Filtered-OFDM (F-OFDM),
2. Filter Bank Multi-carrier (FBMC),
3. Generalized Frequency Division Multiplexing (GFDM), and
4. Universal Filtered Multi-carrier (UFMC).

Different Prototype Filters (PF) for NR systems are described in [6, 7] to minimize the OOBE. PF of wireless systems should have good time-frequency (TF) localization capabilities, particularly in doubly-dispersive channels. Time dispersion increases Inter Symbol Interference (ISI); on the other hand frequency dispersion increases ICI. Each of the waveforms, mentioned above, uses different types of PFs. Different PF and Multi Carrier Modulation (MCM) systems are discussed in [1, 8–13]. FBMC is spectrally efficient [14]. Some reports on FBMC, such as 5GNOW project, PHYDYAS project, have been discussed in [15]. F-OFDM has the flexibility of sub-band splitting [2, 3, 16] and efficient spectrum-utilization through guard band reduction [17]. In case of F-OFDM, soft-truncated Sync Filters are used to truncate infinitely long impulse response of ideal low pass filters. Truncation can reduce the OOBE [18]. Different windows, such as Hann window, Root Raised Cosine (RRC) window, are used for the truncation of Sync Filter [17] of F-OFDM. Proper soft truncation can reduce the OOBE further, thus increasing the quality of wireless channels.

FBMC and UFMC are popular multi-carrier (MC) candidates for wireless communications [19, 20]. MC signals can convert a time dispersive channel into multiplicative channels that can reduce the complexities of the equalizers at the receiver side [21]. FBMC applies filtering technique on each sub-carrier of OFDM. In FBMC based system impulse response of PF has long tail. Therefore Doppler Diversity is less in FBMC based system. On the other hand UFMC based system groups all the sub-carriers in to sub-bands. Each sub-band of UFMC consists of a number of sub-carriers. In UFMC, PF is applied on each sub-band in order to reduce OOBE and achieve higher spectral efficiency [21]. Sub-band based filtering can relax the requirements of global synchronization supporting the inter-subband asynchronous transmission [17]. Dolph-Chebyshev filter is the proposed PF for UFMC [1]. Dolph-Chebyshev filter has constant OOBE level [22]. Even with higher order filter lengths these OOBE remains all most constant, which is not energy efficient. OOBE of Dolph-Chebyshev based UFMC also causes higher level of adjacent band interferences [23–25]. On the other hand Binomial filter produces fewer ripples in the stop band. Though the PAPR is minimum in case of OFDM, however the magnitude of PAPR of different NR waveforms are comparatively higher [26].

Doppler Effect can be minimized by increasing the sub-carrier spacing or by reducing the symbol duration. UFMC is better for low latency services [6] or higher velocity services [1]. UFMC is capable of providing channel adaptive modulations in low-latency applications or vehicular networking. UFMC is also better in Mission Critical Communication (MCM) such as collision avoidance systems, factory automation, and vital sigh monitoring [1].

Future 6g systems will need adaptive wireless links with lower PSD/BER/PAPR in order to minimize interferences and achieve higher bit rate. Wave forms such as FBMC/UFMC/F-OFDM, discussed in different literatures, have the scopes of performance improvement. FBMC/UFMC/F-OFDM systems, proposed in this paper, can perform in a better way in terms of improvement of PSD, BER, OOBE, and PAPR. NR waveforms, proposed in the paper, have better time and frequency localization capabilities in comparison to other NR waveforms. Main contributions of the paper are categorized in ‘three groups of comparisons’ which are mentioned below:

1. Incorporation of FPBF as a porotype window in Filtered-OFDM. FPBF based OFDM (FPBF-OFDM) can show better PSD performance than that of Filtered-OFDM (F-OFDM). Existing F-OFDM based system suffers from poor magnitude response due to wide transition band. As a result existing F-OFDM system is less robust to Multi-User Interference (MUI) due to its higher OOBE [16]. OOBE as well as BER of FPBF- OFDM is better than those of F-OFDM. PAPR of FPBF-OFDM is less than or equal when it is compared with the PAPR of F-OFDM.
2. Incorporation of Binomial filter as a porotype filter in FBMC based systems. Binomial filter based FBMC can show better PSD/OOBE performances than those of PHYDYAS based FBMC. BER of Binomial filter based FBMC is better than that of PHYDYAS based FBMC. PAPR of Binomial filter based FBMC is also less than that of PHYDYAS based FBMC.
3. Incorporation of FPBF as a porotype filter in UFMC based systems. FPBF based UFMC (FPBF-UFMC) can show better PSD performance than that of Chebyshev filter based UFMC. BER of FPBF-UFMC is better than that of Chebyshev filter based UFMC. PAPR of FPBF-UFMC is also less than or equal when it is compared with the PAPR of Chebyshev filter based UFMC.

## Different proto type filters for filtered-OFDM

**Fig 1:**
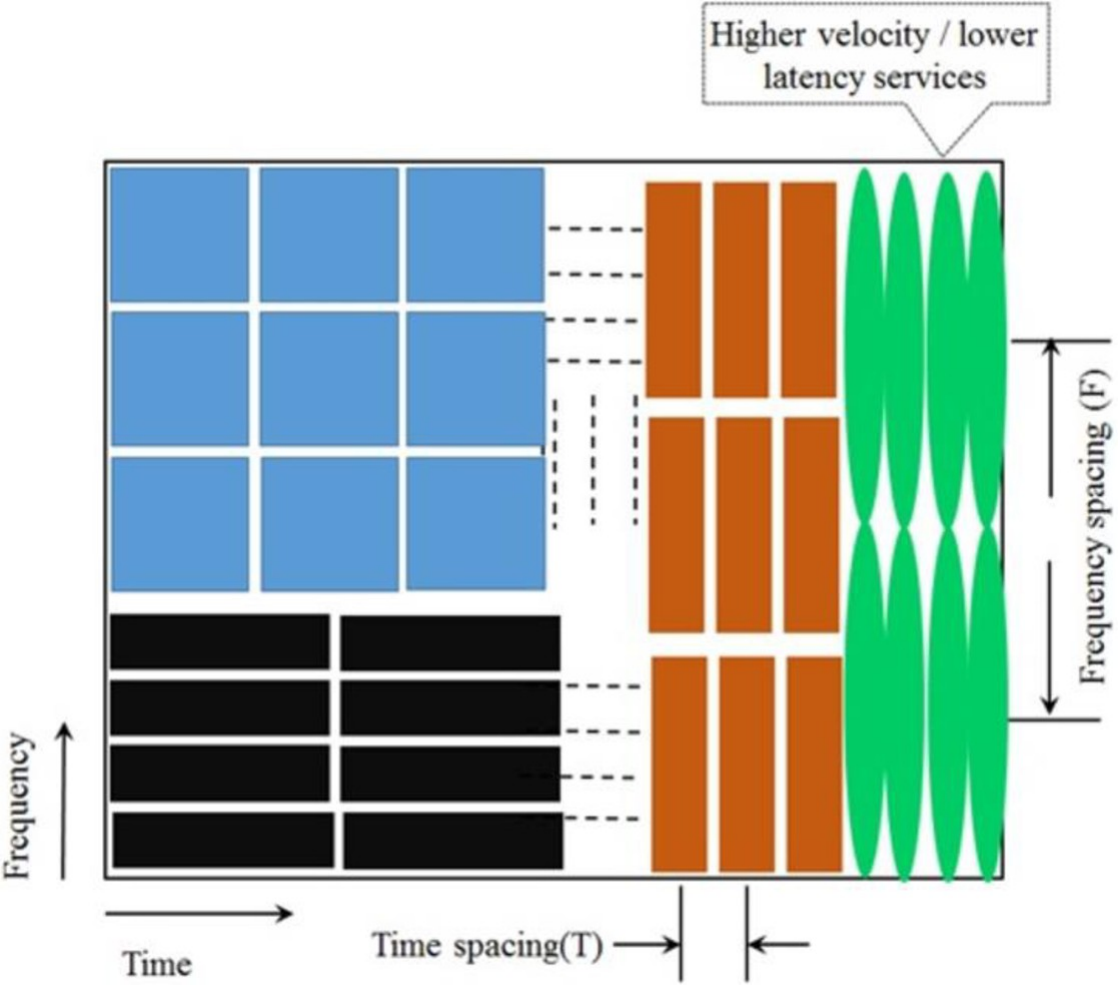
Different time-frequency allocation of sub-carriers in wireless air interface.

**Fig 2:**
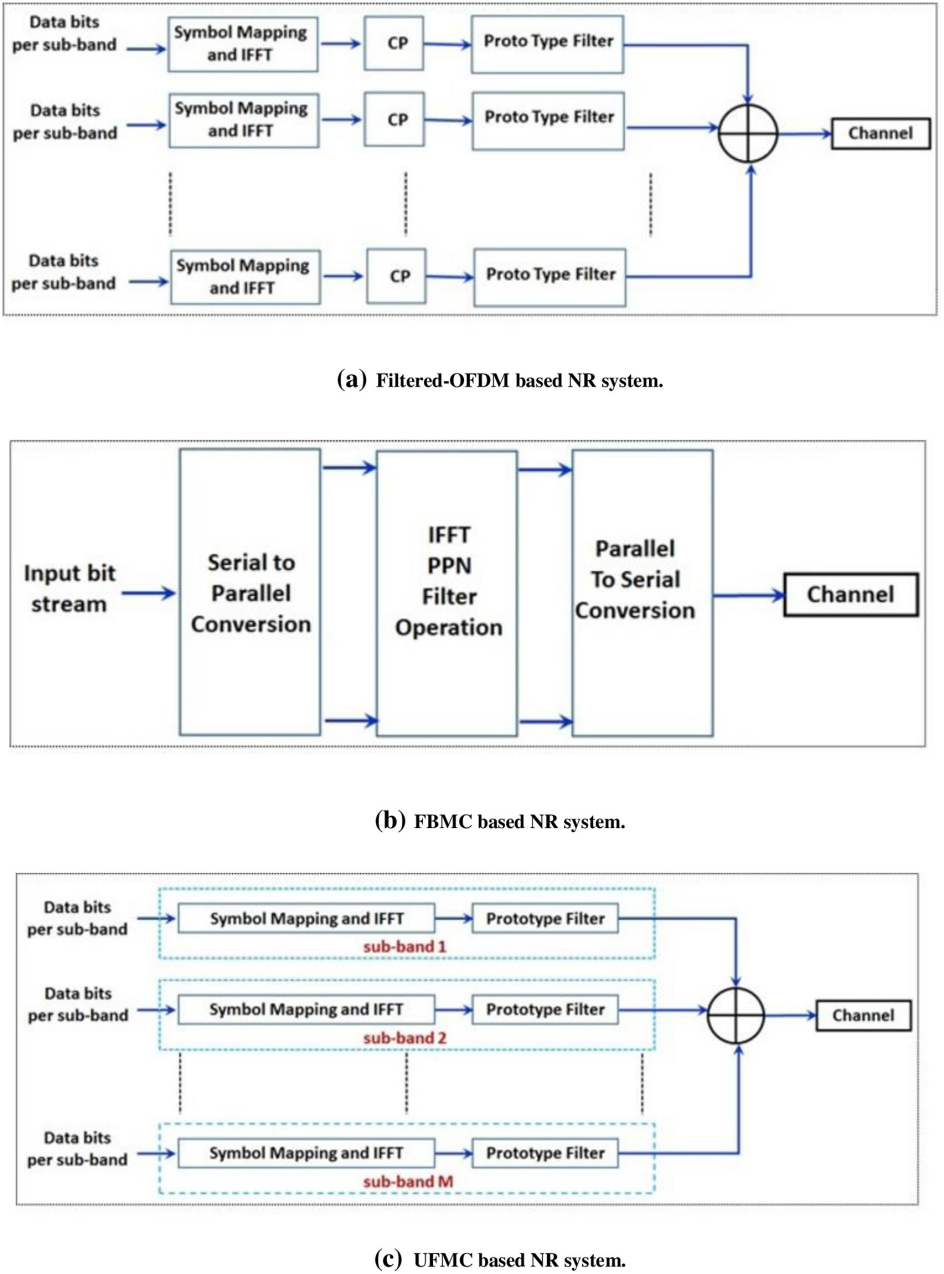
Block diagrams of different NR interfaces at the transmitter sides. (a) Filtered-OFDM based NR system. (b) FBMC based NR system. (c) UFMC based NR system.

It is mentioned in the previous section that PF of NR systems should have fine Time-Frequency (TF) localization capabilities. Appropriate TF localization of the Resource Blocks (RBs) is an essential characteristic of wireless systems. Particularly better TF localization is needed in case of doubly-dispersive channels. Fig 1 represents an example of TF allocations for different sub-carrier systems. Fig 2 illustrates the block diagrams of three different NR interfaces at the transmitter sides:

1. Filtered-OFDM (F-OFDM),
2. Filtered Bank Multi Carrier (FBMC), and
3. Universal Filter Multi Carrier (UFMC).

Several MCM approaches addressing different 5G technical issues can be found in the literatures [18]. Performances of three different NR waveforms are compared in the following sub-sections and sections considering the performance parameters PSD, BER, PAPR, and SIR. Following sub-sections describe a novel and better method of filtering/windowing for F-OFDM based systems. Subsequent sections describe improved method of filtering for FBMC using Binomial filter and UFMC based systems using FPBF.

### Fractional Powered Binomial Filter (FPBF) for F-OFDM based waveform

RBs of F-OFDM can be flexibly grouped depending upon the traffic profile, loading conditions, and QoS. Generally the PF of F-OFDM suffers from poor magnitude response due to wide transition band [27]. As a result existing F-OFDM system is less robust to Multi-User Interference (MUI) due to its higher OOBE [16]. In this sub-section a novel and better method of filtering/windowing for OFDM using Fractional Powered Binomial Filter (FPBF) is described. Transmitted signal for the proposed FPBF-OFDM can be expressed using (1):

$$\tilde{s}\left(m\right)=s\left(m\right)*f_{FPBF}\left(m\right)$$ (1)

where

$$f_{FPBF}\left(m\right)=f_{sync}\left(m\right).C_{FPBF}$$ (2)

*f*_*FPBF*_(*m*) = FPBF based truncated sync filter (window);

*f*_*sync*_(*m*) = Time domain sync filter;

$$s\left(m\right)=\Sigma_{k=0}^{K-1}s_{k}\left(m-k\left(M+Ng\right)\right)$$ (3)

[2]

M = IFFF/FFT length;

K = No. of OFDM symbols;

*N*_*g*_ = CP length;

CFPBF=[bL]ρ=aLΣL=0NaLρ= (4)

*Fractional Powered Binomial Filter*;

$$a_{L}=\frac{N!}{L!\left(N-L\right)!}\;\;L=0,1\ldots \;,N;$$ (5)

bL=aLΣL=0QaL (6)

In (4) the value of *ρ* is a variable within the range 0 ≤ *ρ* ≤ 1. Coefficients of (4) are derived from Binomial coefficients and hence the filtering technique using (4) is named as FPBF.

### Performance comparison of F-OFDM and FPBF-OFDM

**Fig 3:**
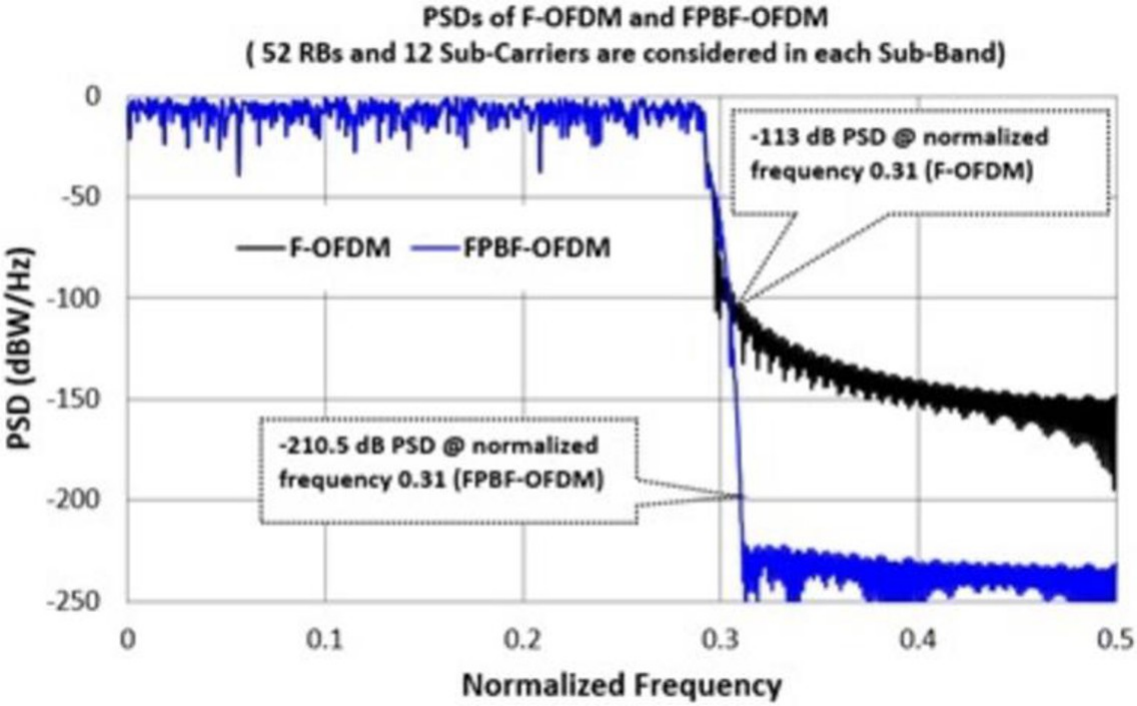
Power spectral densities (PSDs) of F-OFDM and FPFB-OFDM.

It was mentioned before that existing F-OFDM shows poor performance due to wide transition band [27]. FPBF based OFDM performs better than F-OFDM systems particularly when PSDs are compared. Fig 3 shows the PSD of FPBF-OFDM and F-OFDM for 52 resource blocks (RBs). Each RB consists of 12 Sub-Carriers. Other parameters of FPBF-OFDM/F-OFDM are summarized in Table 1. It can be observed from Fig 3 that the reduction of OOBE in case of FPBF-OFDM is much faster/lower than that of F-OFDM.

**Table 1:** Simulation parameters for different waveforms.

**Overall Parameters**		**Type of New Radio (NR) Waveform**									
**QAM-order**	**Size of RB**	**F-OFDM**		**FPBF-OFDM**			**UFMC**			**FBMC**	
		**CP Length**	**Filter Length**	**CP Length**	**Sync Filter Length**	**FPBF Window Length**	**PF: Dolph-Chebyshev**		**PF: FPBF**	PF: PHYDYAS	PF: Binomial Filter
							**Filter Length**	**Side Lobe Attenuation**	**Filter Length**		
64/ 256	12	72	513	72	513	513	513	40	513		

**Fig 4:**
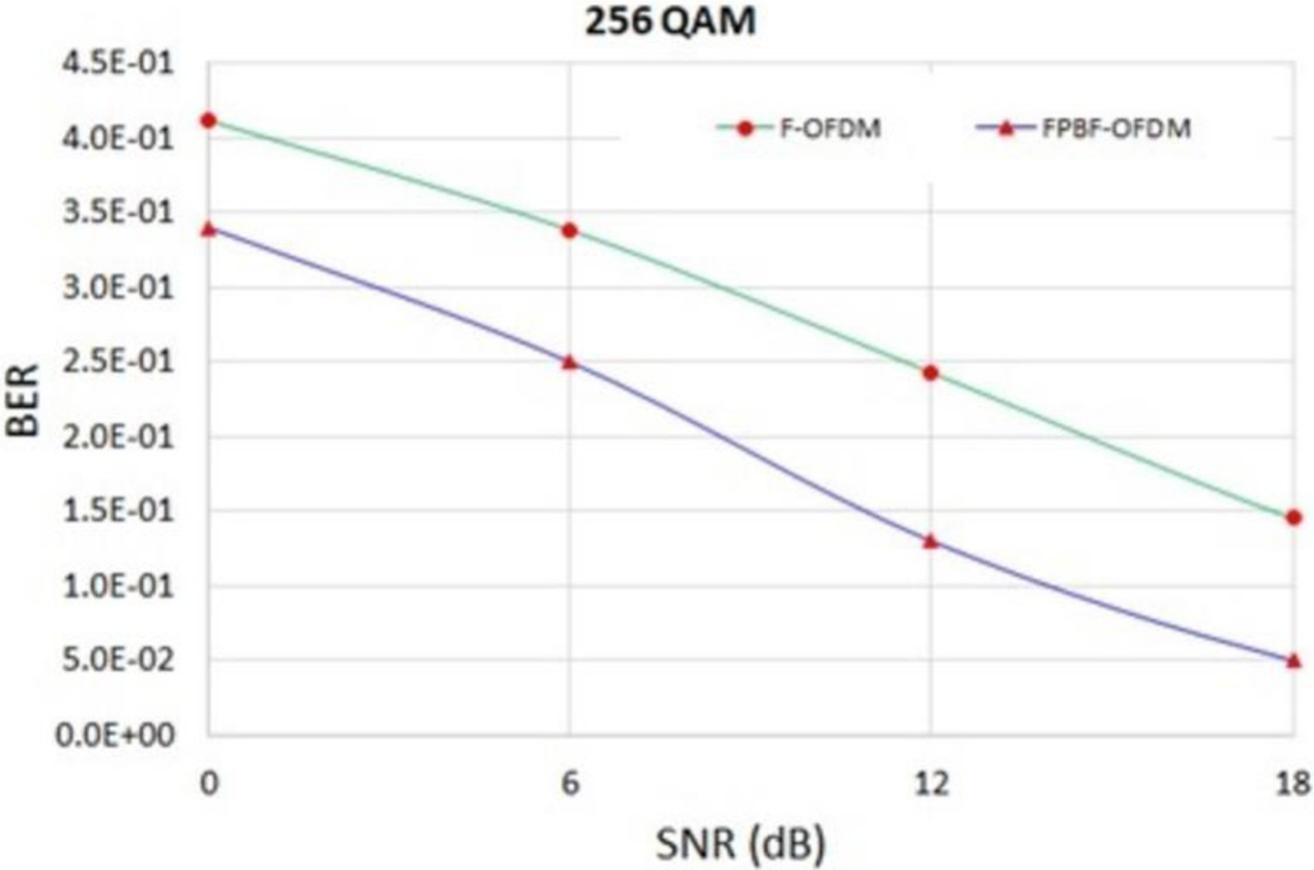
BER of F-OFDM and FPFB-OFDM for different SNR level.

**Fig 5:**
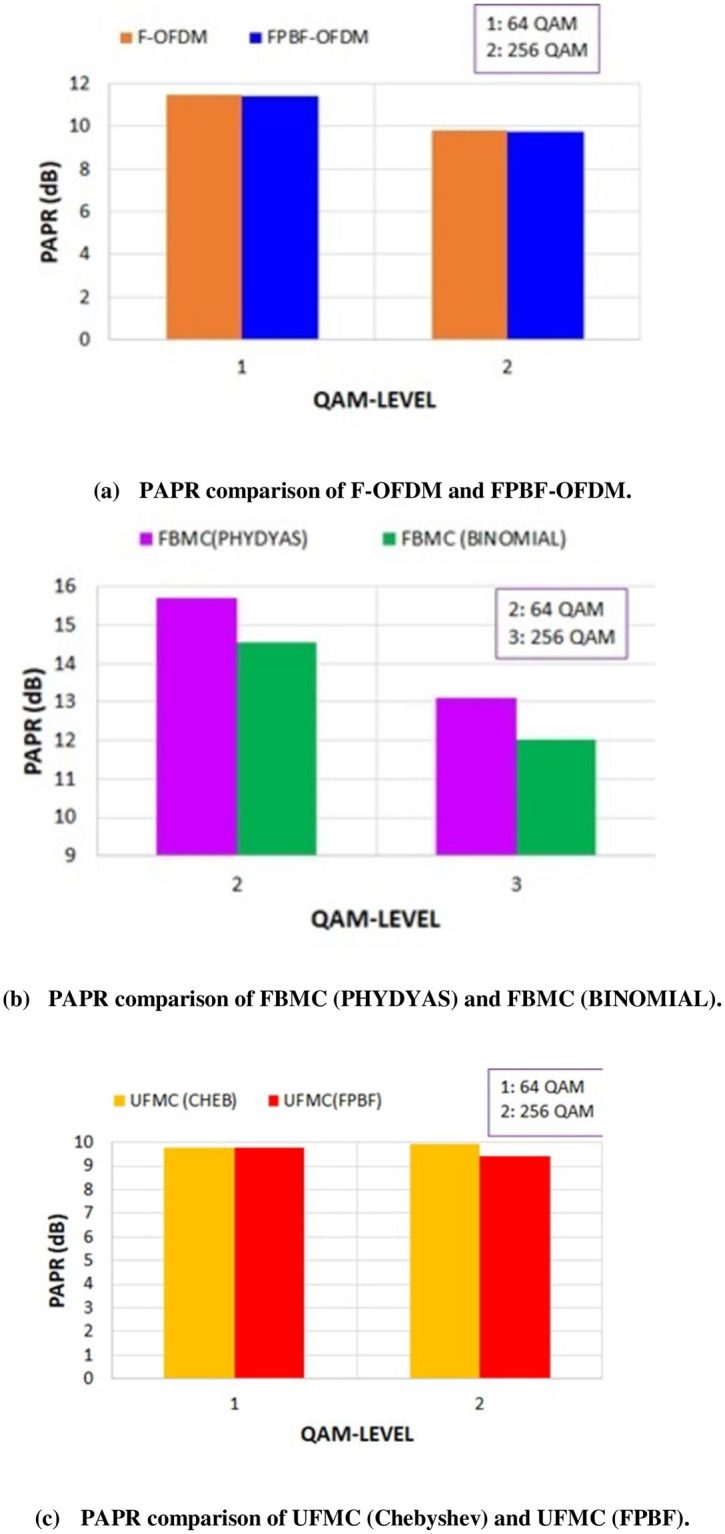
PAPR of different NR systems with different QAM levels. (a) PAPR comparison of F-OFDM and FPBF-OFDM. (b) PAPR comparison of FBMC (PHYDYAS) and FBMC (BINOMIAL). (c) PAPR comparison of UFMC (Chebyshev) and UFMC (FPBF).

OOBE is -113 dB for F-OFDM and at the normalized frequency position of 0.31. On the other hand, OOBE is -210.5 dB for FPBF-OFDM and at the same normalized frequency. This improvement of OOBE in case of novel FPBF-OFDM will reduce the ICI further. BER in different QAM/SNR scenarios of FPBF-OFDM were also found better than those of F-OFDM. Fig 4 shows the BER of FPBF-OFDM/F-OFDM for SNR range 0–18 dB. Fig 5 shows the PAPR of different NR systems at two different QAM levels, 64-QAM and 256 -QAM. Figs 3–5 depict that novel FPBF-OFDM can perform better than existing F-OFDM when PSD, BER and PAPR performances are compared.

## Proposed New Radio (NR) waveform for FBMC

Block diagram of a FBMC based transmitter is shown in Fig 2(b). Transmitted signal for FBMC based NR waveform can be expressed as (7):

xt=ΣnN−1Σk=0sknsknpkt−nT2 (7)

where,

$$P_{k}\left(t\right)=p\left(t\right)e^{jkFt}e^{j\left(k+n\right)/2},$$

*s*_*k*_ is the subcarrier data symbol, *k* is the subcarrier index,

*T* is the symbol period, and

p(t) is the prototype filter of FBMC.

In FBMC system, a group of parallel data symbols s_k_[n] is transmitted across an analysis filter bank.

Though frequency-localization capability of PHYDYAS PF based FBMC is better, however it needs to sacrifice time-localization in order to achieve optimum TF localization which can be inferred from Fig 1. Due to this issue BER of PHYDYAS based FBMC becomes higher. PAPR of PHYDYAS based FBMC is also higher. Higher PAPR causes non-linearity issues in power amplifiers. The issues of higher BER/PAPR can be minimized using Binomial filter in FBMC. Methods of signal processing through efficient Binomial window/filter are described in [28, 29]. Construction of Binomial window/filter is based on the Binomial coefficients of (6). Performances of FBMC with Binomial and PHYDYAS filters are compared in the following sub-section.

### Performance-comparison of FBMC with Binomial and PHYDYAS filters

**Fig 6:**
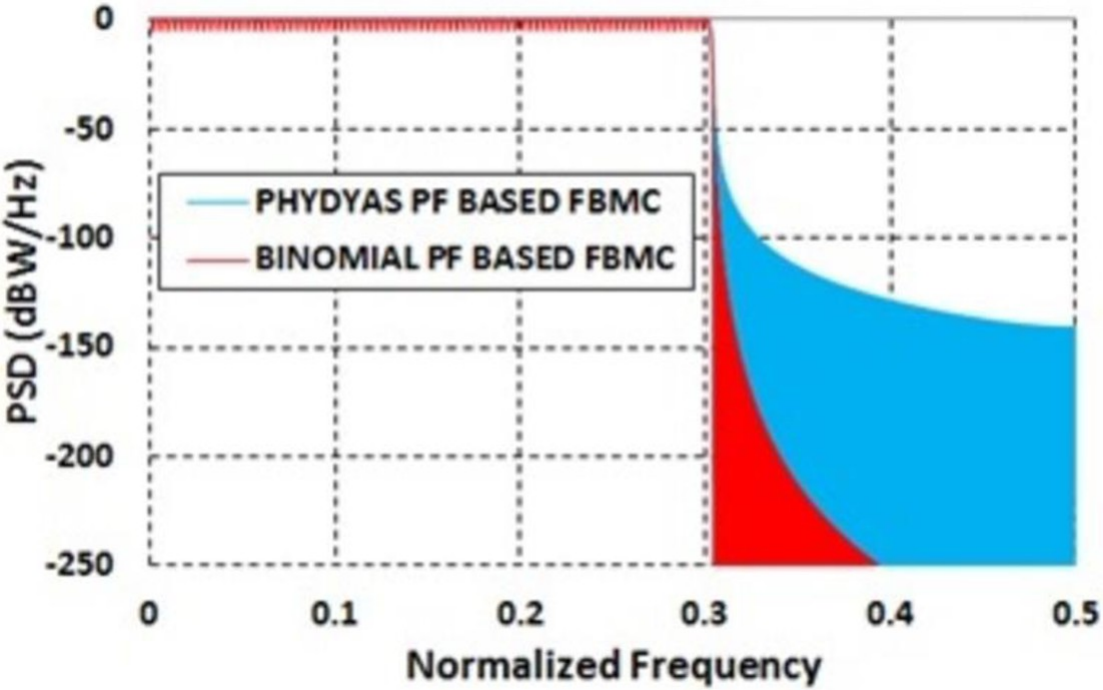
Power spectral densities of PHYDYAS based FBMC and Binomial filter based FBMC.

**Fig 7:**
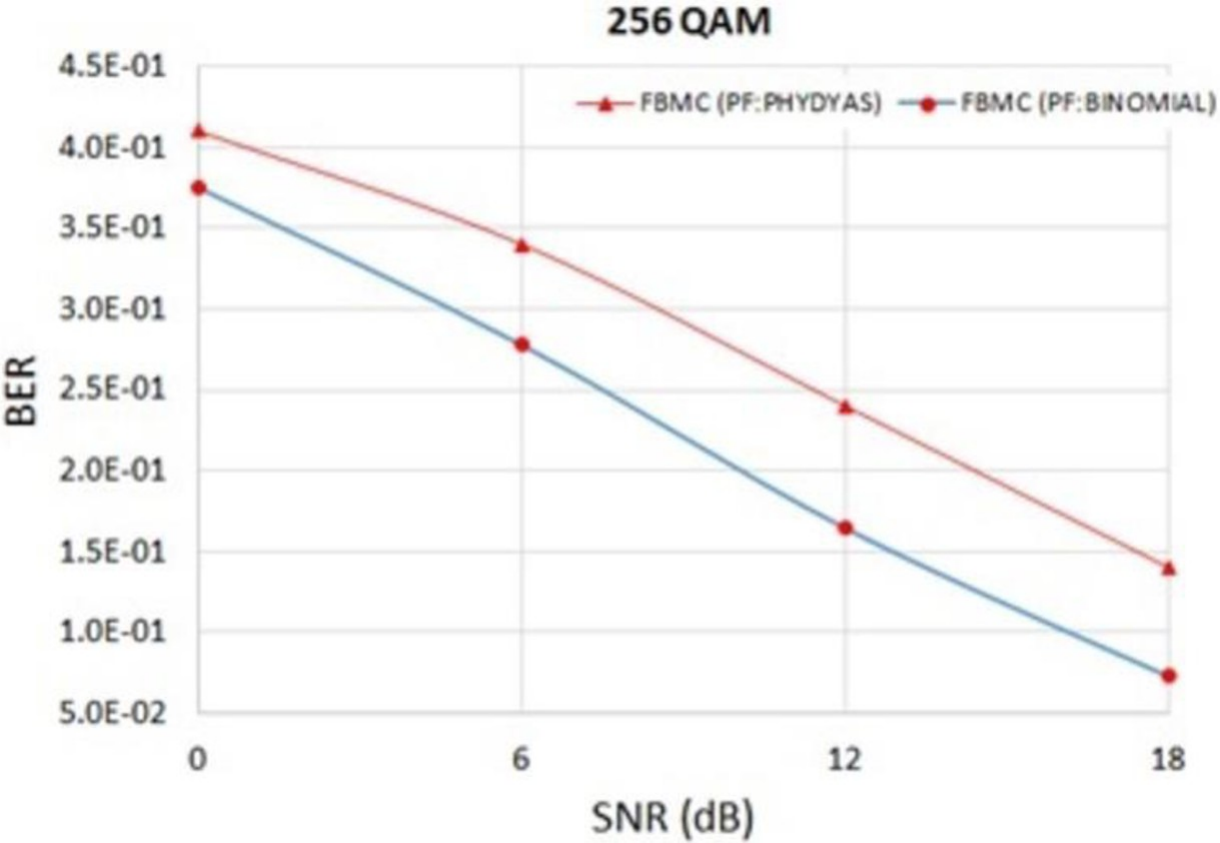
BER comparison at different SNR with Binomial and PHYDYAS filters of FBMC.

Fig 6 shows the PSDs of FBMC with two different prototype filters, one is with Binomial filter and another one is with PHYDYAS filter. It can be observed from Fig 6 that the reduction of OOBE is much better in case of Binomial filter. OOBE at normalized frequency 0.31 is -60.4 dB in case of Binomial filter. On the other hand, OOBE is -40.7 dB in case of PHYDYAS filter. It was mentioned before that FBMC based method found in the literatures suffers from higher PAPR problem [30]. Fig 5(b) shows that the PAPR in case of Binomial filter is lower than that of PHYDYAS filter. BER at different SNR is also lower in case of Binomial filter (Fig 7).

## Proposed Fractional Powered Binomial Filter (FPBF) for UFMC

It was mentioned before that in case of UFMC the whole frequency band is divided into sub-bands and a PF is applied on each sub-band. Different literatures have proposed Dolph-Chebyshev PF for UFMC [1, 21, 24]. OOBE in case of Dolph-Chebyshev PF causes higher inter sub-band interferences [23, 24]. A UFMC based transmitter block diagram is shown in Fig 2(c). Time domain signal vector *x*_*k*_ for the user k in UFMC transmission scheme can be expressed as:

$$x_{k}=\Sigma_{i=0}^{M}x_{i,k}*w_{i,k}$$ (8)

Where,

M is total number of sub-bands;

*x*_*i*,*k*_ is the transmitted data vector within sub-band i after applying N point IFFT;

*w*_*i*,*k*_ is the proto-type filter applied on sub-band I;

In this section the novel and better FPBF (4) based PF is investigated for UFMC. It is noteworthy to mention here that (4) is also investigated in the previous section for the windowing of filtered OFDM. In case of FPBF-OFDM filtering is performed on each sub carrier. On the other hand filtering operation of propose FPBF based UFMC is done one each sub-band [25]. UFMC based transmitted signal (8) using FPBF can be expressed as (9):

$$x_{k}=\Sigma_{i=0}^{M}x_{i,k}*C_{FPBF}\left(i,k\right)$$ (9)

Calculation process of *C*_*FPBF*_ is already described in previous section.

**Fig 8:**
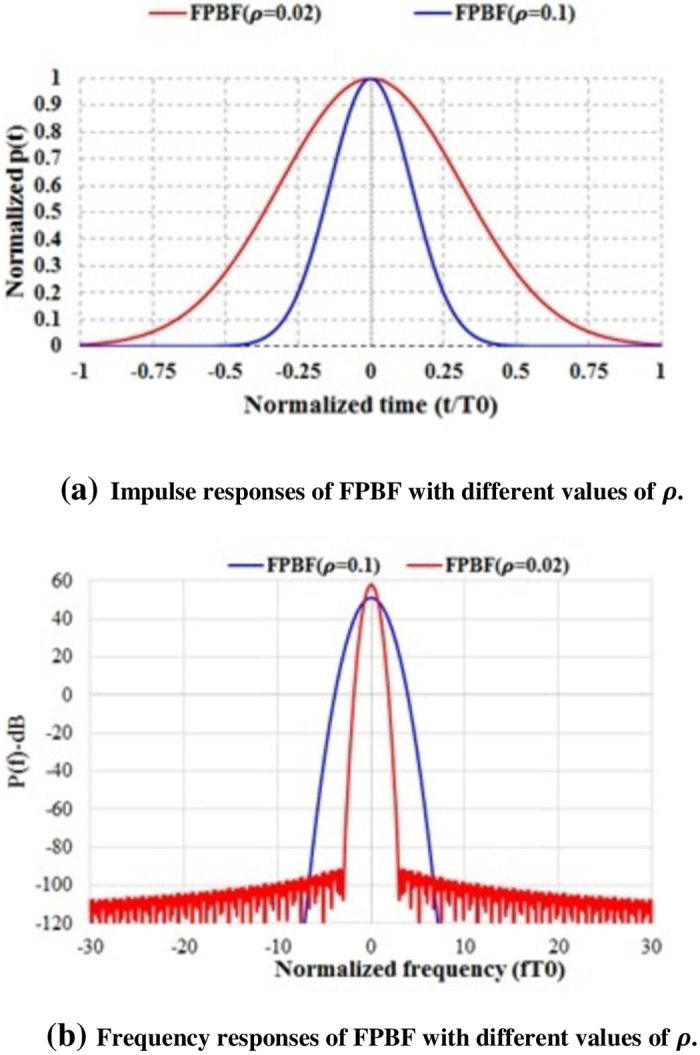
Impulse responses (IR) and frequency responses (FR) of Fractional Power Binomial Filter with different values of *ρ*.

Fig 8 shows the impulse responses and frequency responses of FPBF for different values of ***ρ***. In case of FPBF based UFMC, the time-frequency tiles, as shown Fig 1, can be controlled adaptively by choosing an appropriate value of ***ρ***.

### Performance comparison of FPBF based UFMC and Dolph-Chebyshev filter based UFMC

**Fig 9:**
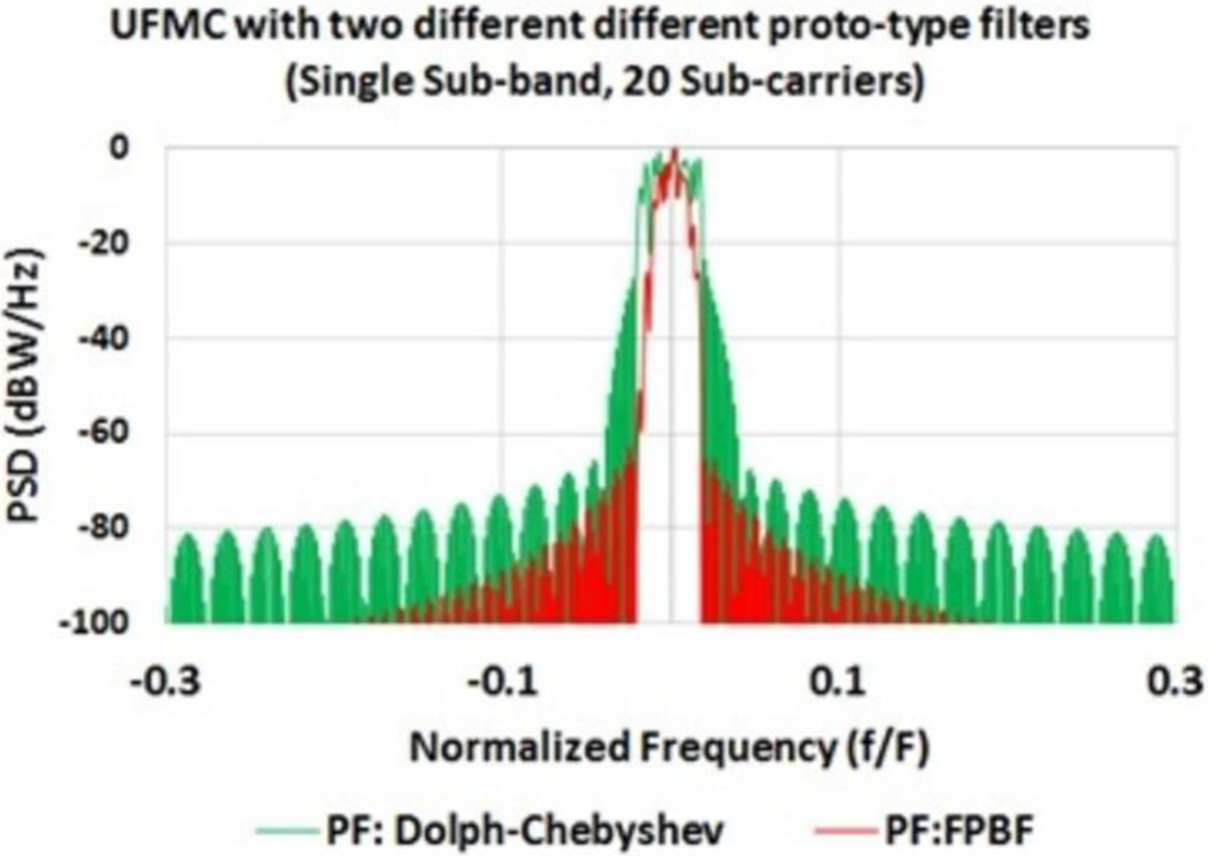
PSD of FPBF based UFMC and Dolph-Chebyshev filter based UFMC.

**Fig 10:**
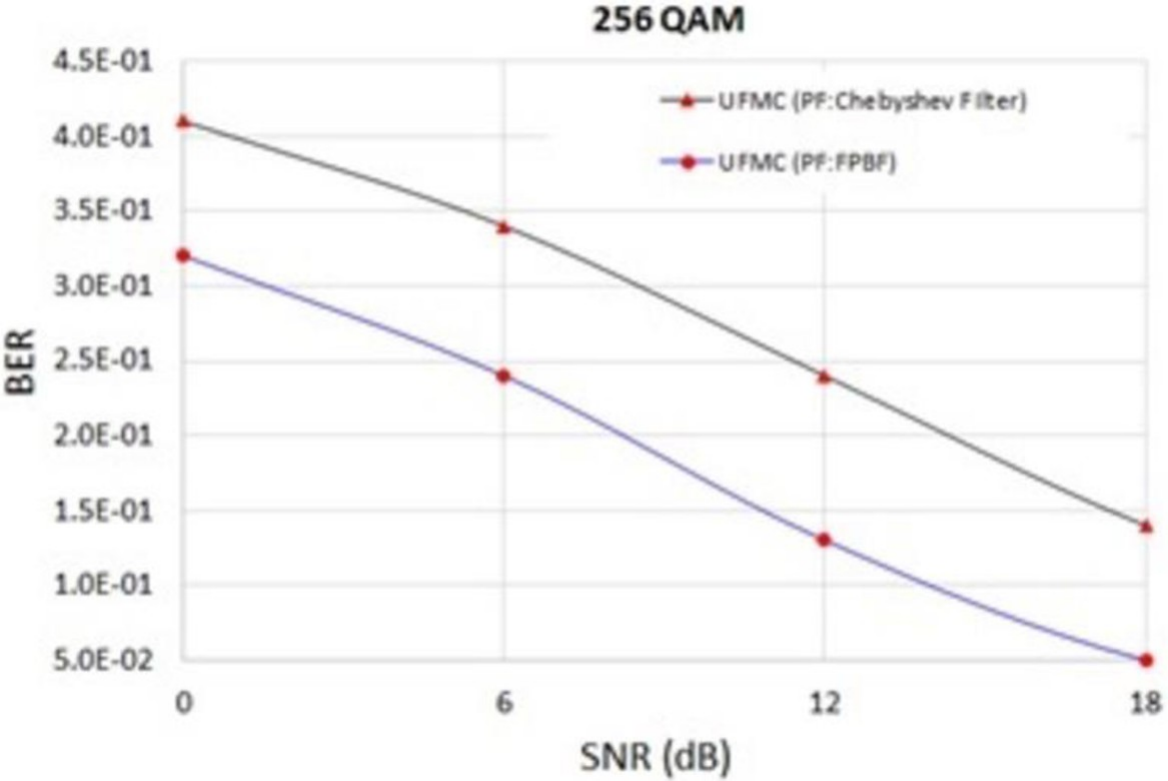
BER of FPBF and Chebyshev filter based UFMC at different SNR.

It was found that the PSD of FPBF based UFMC is better than that of Dolph-Chebyshev filter based UFMC (Fig 9). Fig 9 shows the PSDs of one sub-band out of 52 simulated sub-bands (each sub-band contains 20 sub-carriers). It can be observed from Fig 9 that the inter sub-band interferences are minimized in case of FPBF based UFMC. Interference level within 3^rd^~52^th^ sub-bands (not shown in the figure), due to 1^st^ sub-band, was about 122 dB less in case of FPBF-UFMC in comparison to that of Dolph-Chebyshev based UFMC. In this case the value of ***ρ*** was chosen 0.045 to achieve optimum performance. In real scenarios ***ρ*** can be chosen adaptively or using any advanced signal processing algorithm. BER performances of FPBF based UFMC are also comparatively better (Fig 10). FPBF based UFMC can also improve the Doppler Diversity [31].

### Improvement of Signal to Interference Ratio (SIR) of UFMC

Concise Time-Frequency localization in time-frequency tiles is essential for the mitigation of Doppler Effect. Duration of the transmitted symbols is needed to be perfectly matched with the duration of Impulse response of the PF in time critical applications. Upper bound of ICI depends on the maximum Doppler Effect (*f*_*d*_) and duration of the transmitted symbol ((*T*_*s*_). Therefore, to reduce the ICI due to Doppler Effect, *f*_*d*_*T*_*s*_ should be made minimum (i.e. some waveform parameters, such as sub-carrier spacing, should be chosen according to the wireless channel statistics. Some of the channel statistics areDoppler effect anddelay spread.

In 5G systems, different sub-carrier spacing (SCS) are used (such as 15 KHz, 30 KHz, 60 KHz and 120 KHz). It was mentioned before that UFMC is better for low latency services [1] or higher velocity applications [3]. SCS of FPBF based UFMC can be utilized in a better way than the SCS of Dolph-Chebyshev based UFMC.

**Fig 11:**
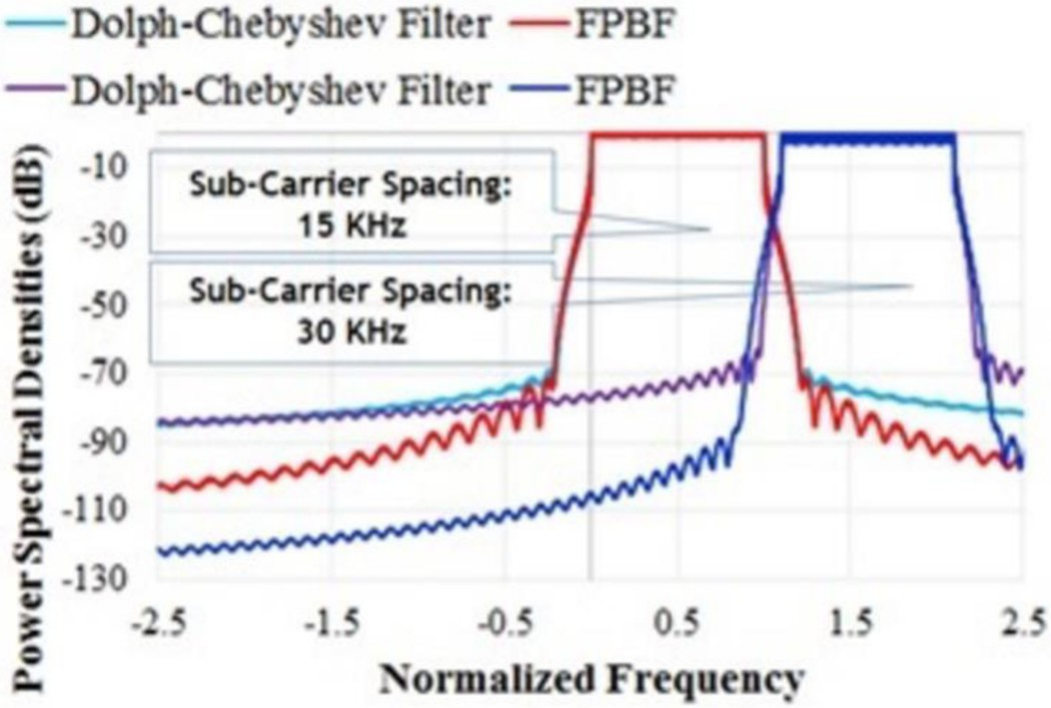
PSD of UFMC for two different sub-carrier spacing with FPBF and Chebyshev filter.

In this sub-section two different SCS of UFMC (15 KHz and 30 KHz) are considered for two users in order to investigate the effect of proto-type filters [1]. Fig 11 shows the PSDs of UFMC for two different SCS using FPBF and Dolph-Chebyshev Filter. It can be observed from Fig 11 that the inter sub-band interference is lower in case of FPBF based UFMC. Some of the simulation parameters and results for the cases are summarized in Table 2.

**Table 2:** Simulation parameters for different sub-carrier spacing of UFMC for SIR estimation (Parameters for UFMC based NR with two different proto-type filters).

**Prototype Filter**		**SCS (KHz)**		**QAM Level**	**FFT Size**
	**Sub-Carrier Spacing (KHz)**	15	30	256	512
	**No. of sub-carriers in each sub-band**	96	48		
	**Total bandwidth (MHz)**	1.44	1.44		
**Fractional Power Binomial Filter (*β* = .05)**	**SIR in dB (guard band 0.3)**	61.64	67.53		
**Dolph-Chebyshev Filter (attenuation 40 dB)**		56.37	50.98		

**Fig 12:**
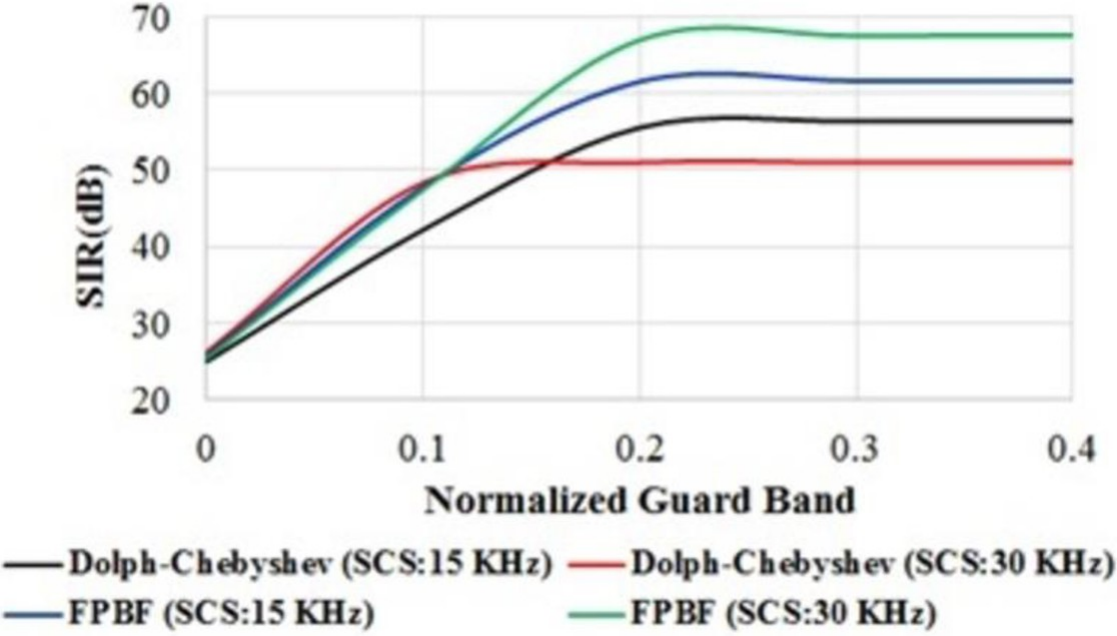
PSD of UFMC for two different sub-carrier spacing with FPBF and Chebyshev filter.

SIR within each sub-band, one with 15 KHz SCS and the other with 30 KHz SCS, was also investigated. Fig 12 shows the SIR for two different proto-type filters with 15 KHz/30 KHz SCS. Calculation process of SIR can be found in [3]. It can be observed from Fig 12 and Table 2 that FPBF can enhance the SIR of UFMC for different sub-carrier spacing. For example, the SIR at 15 KHz SCS with Dolph-Chebyshev filter is 56.37 dB at the normalized guard-band of 0.3. On the other hand the SIR of identical case of FPBF is 61.64 dB. SIR improvement of 5.27 dB is observed with FPBF. Similarly, the SIR with 30 KHz SCS and 0.3 guard-band are 67.53 dB and 50.98 dB respectively for the FPBF and Dolph-Chebyshev filter. In this case also an SIR improvement of 16.55 dB is observed with FPBF. Enhancement of SIR with prototype FPBF will increase the Doppler Diversity in UFMC which is suitable for low latency applications.

## Conclusion

One of the main components of New Radio (NR) technologies is the Prototype Filter (PF). PF plays an important role in performance improvement of different wireless communication systems. Different NR waveforms and PF are compared in this paper. PSD, BER, PAPR, and SIR of different waveforms are compared as performance indicators. Performance parameters are compared in three major categories which are summarized below-

1. **FPBF-OFDM vs. F-OFDM in case of filtered OFDM**:
  PSD improvement in case of FPBF-OFDM is 97.5 dB at the normalized frequency of 0.31 and BER improvement is 0.07 at 0 dB SNR.
2. **Binomial filter and PHYDYAS filter in case of FBMC**:
  OOBE improvement in case of Binomial filter is 19.7 dB at the normalized frequency of 0.31 and BER improvement is 0.03 at 0 dB SNR. PAPR improvement in case of Binomial filter is 1.16 dB with 64-QAM and 1.1 dB with 256-QAM.
3. **FPBF and Dolph-Chebyshev filter in case of UFMC**:
  Improvement of interference level is 122 dB within 3^rd^~52^th^ sub-bands due to 1^st^ sub-band of FPBF base UFMC. BER improvement of FPBF based UFMC is 0.07 at 0 dB SNR. SIR improvement of FPBF-UFMC is 5. 27 dB with 15 KHz sub-carrier spacing and 16.55 dB with 30 KHz sub-carrier spacing.

It can be concluded from the above comparisons that FPBF-OFDM, Binomial filter based FBMC, and FPBF based UFMC are better than their counter parts described in the paper. Results with different NR prototype filters, discussed in the paper, are summarized in Table 3. Applications of F-OFDM in Internet of Things (IoT) as well Machine Type Communications (MTC) are discussed in [32]. Proposed FPBF-OFDM would be able to show better performances in different IoT and MTC applications. Proposed Binomial filter Based FBMC can be used in Digital Video Broadcasting (DVB) since Binomial filter based FBMC will meet the performance requirements of DBV in a better way. Applications of UFMC in Machine to Machine and car-to-car communications are discussed in [33]. Since the performances of FPBF based UFMC is better than those of Chebyshev filter based UFMC, the proposed FPBF based UFMC can be applied to Machine to Machine as well as car-to-car communications. Finally it can be concluded that the novel and better proto type filters for different New Radio (NR) waveforms, discussed in the paper, are the good candidates for future 6G wireless systems.

**Table 3:** Performance comparison of different New Radio (NR) waveforms.

Types of New Radio (NR) waveform	Prototype Filter	PSD	BER	OOBE/ Interference	PAPR/SIR
**Filtered OFDM**	**Novel FPBF**	97.5 dB improvement with FPBF	Improvement of 0.07 with FPBF @ 0 dB SNR	X	PAPR are almost equal in FPBF-OFDM and F-OFDM
	**F-OFDM**				
**FBMC**	**Novel Binomial**	X	Improvement of 0.03 with Binomial filter @ 0 dB SNR	OOBE improvement of 97.5 dB with Binomial filter	PAPR improvement of 1.1 dB with Binomial filter @ 256-QAM
	**PHYDYAS**				
**UFMC**	**Novel FPBF**	X	Improvement of 0.07 with FPBF @ 0 dB SNR	Reduction of 122 dB interference with FPBF.	SIR improvement of 16.55 dB with FPBF @ 30 KHz sub-carrier spacing. PAPR is less than or equal to in case of FPBF.
	**Chebyshev filter**				

## Supporting information

S1 Data (RAR)
